# Factors influencing maternal nutrition practices in a large scale maternal, newborn and child health program in Bangladesh

**DOI:** 10.1371/journal.pone.0179873

**Published:** 2017-07-10

**Authors:** Phuong H. Nguyen, Tina Sanghvi, Sunny S. Kim, Lan M. Tran, Kaosar Afsana, Zeba Mahmud, Bachera Aktar, Purnima Menon

**Affiliations:** 1 Poverty, Health, and Nutrition Division, International Food Policy Research Institute, Washington, DC, United States of America; 2 Alive & Thrive, Washington, DC, United States of America; 3 BRAC, Dhaka, Bangladesh; McMaster University, CANADA

## Abstract

Improving maternal nutrition practices during pregnancy is essential to save lives and improve health outcomes for both mothers and babies. This paper examines the maternal, household, and health service factors influencing maternal nutrition practices in the context of a large scale maternal, newborn, and child health (MNCH) program in Bangladesh. Data were from a household survey of pregnant (n = 600) and recently delivered women (n = 2,000). Multivariate linear and logistic regression analyses were used to examine the determinants of three outcomes: consumption of iron and folic acid (IFA) tablets, calcium tablets, and diverse diets. Women consumed 94 ± 68 IFA and 82 ± 66 calcium tablets (out of 180 as recommended) during pregnancy, and only half of them consumed an adequately diverse diet. Good nutrition knowledge was the key maternal factor associated with higher consumption of IFA (β = 32.5, 95% CI: 19.5, 45.6) and calcium tablets (β ~31.9, 95% CI: 20.9, 43.0) and diverse diet (OR = 1.8, 95% CI: 1.0–3.1), compared to poor knowledge. Women’s self-efficacy in achieving the recommended practices and perception of enabling social norms were significantly associated with dietary diversity. At the household level, women who reported a high level of husband’s support were more likely to consume IFA (β = 25.0, 95% CI: 18.0, 32.1) and calcium (β = 26.6, 95% CI: 19.4, 33.7) tablets and diverse diet (OR = 1.9, 95% CI: 1.2, 3.3), compared to those who received low support. Health service factors associated with higher intakes of IFA and calcium tablets were early and more prenatal care visits and receipt of free supplements. Combined exposure to several of these factors was attributed to the consumption of an additional 46 IFA and 53 calcium tablets and 17% higher proportions of women consuming diverse diets. Our study shows that improving knowledge, self-efficacy and perceptions of social norms among pregnant women, and increasing husbands’ support, early registration in prenatal care, and provision of free supplements will largely improve maternal nutrition practices.

## Introduction

Maternal undernutrition, including macro- and micronutrient deficiencies, is a significant public health problem in many developing countries, especially in South Asia [1,2]. An estimated 32 million pregnant women (38%) globally are anemic, with the second highest anemia prevalence at 52% in the South Asia region [3]. The proportion of undernourished women of reproductive age with body mass index (BMI) of less than 18.5 kg/m^2^ is also very high in South Asia, at more than 20% [4]. Maternal undernutrition is a major concern because of its association with mortality and overall disease burden for both mothers and their children [4].

Improving the delivery of maternal nutrition interventions is important to reduce the high burden of maternal and child undernutrition and mortality [5]. Evidence-based interventions during the critical pre-pregnancy to conception periods of life include nutrition counseling to improve the quantity and diversity of foods consumed and balanced energy-protein intake; supplementation with iron and folic acid (IFA), calcium, and other micronutrients; and, in some contexts, food fortification [6,7]. However, scaling up access to these interventions and increasing their utilization pose a large challenge, particularly in high burden countries [8].

A systematic review of large-scale maternal nutrition programs in several countries showed mixed findings in terms of implementation issues [9]. Food fortification with IFA was less successfully scaled up than salt iodization initiatives. In Nepal and Nicaragua, micronutrient supplementation programs achieved good coverage under conditions of high antenatal care coverage, availability of tablets, and compliance. However, programs that integrated food supplementation and behavioral change interventions in India, Bangladesh, and Madagascar achieved only moderate coverage [9]. A recent study of barriers to providing IFA supplementation through antenatal care in 22 countries [8] identified four ‘falter points’ to delivering at scale: 1) low antenatal care coverage, 2) irregular supplies of IFA tablets, 3) low acceptance of IFA, and 4) low adherence to 180 days of supplementation. Addressing these falter points is a necessary step towards improving program effectiveness. Although multiple studies have examined factors associated with IFA receipt or adherence including personal, socio-cultural, or logistical factors [9–11], few have studied the factors associated with calcium supplementation, which is a newly recommended intervention during pregnancy [12]. There is also scarce information on factors influencing utilization of nutrition education and counseling on dietary diversity.

Despite improvements in several health and development indicators in recent years, Bangladesh remains one of the countries with the highest prevalence of maternal undernutrition. Nearly a quarter of women of reproductive age are undernourished or underweight (BMI <18.5kg/m^2^) [13], and one-half of all pregnant women are anemic [14], mostly due to iron deficiency. Calcium intake is also low, with two-thirds of women consuming 200 mg of calcium or less [15,16], as compared with the recommended level of 1000 mg per day [17], placing pregnant women at higher risk of hypertensive disorders and eclampsia/pre-eclampsia [4].

To address these challenges, a large national non-governmental organization in Bangladesh (BRAC) intended to integrate intensified maternal nutrition interventions into its existing community-based maternal, neonatal, and child health (MNCH) program. This paper examines the maternal, household, and health service factors that influenced maternal nutrition practices in the MNCH program areas; and highlights the key factors that, if strengthened, could markedly improve practices in this context.

## Methods

### Program description

The MNCH program started in 2010 and currently operates in 14 districts and covers 24.9 million mothers and children [18]. BRAC frontline health workers (FHWs) conducted monthly home visits to provide services for mothers including family planning, identification of pregnancies, prenatal, delivery and postnatal care, essential neonatal care, management of neonatal and child illnesses, promoting vaccination, referral for complications and improving the access to clinical services in health facilities. The standard nutrition interventions delivered through the MNCH program include nutrition education, selling of IFA (60 mg iron and 400 μg folic acid) and calcium (500 mg) supplements to pregnant women, deworming for women, and counseling on infant and young child feeding practices. Women are also able to receive IFA for free if they seek prenatal care at government clinics. National policy guidelines currently recommend the consumption of IFA (60 mg iron and 400 μg folic acid) and calcium (1200 mg) tablets daily for 6 months, or approximately 180 tablets, during pregnancy. A lower dosage of calcium supplements is provided in the MNCH program, based on recent research on lower dosage [19] and due to complementary activities to promote dietary diversity. The MNCH program has achieved substantial gains in access to family planning and antenatal care [18], but there is low utilization of maternal nutrition interventions [16].

### Data sources and study population

The data source for this paper was the baseline household survey conducted in 2015 as part of an evaluation to test the feasibility and impacts of integrating intensified maternal nutrition interventions into the existing MNCH program platform in Bangladesh (registered at ClinicalTrials.Gov as NCT02745249). The survey was carried out in 20 rural sub-districts (*upazilas*) from four districts (Mymensingh, Rangpur, Kurigram, and Lalmonirhat) where the MNCH program has been in place for more than 5 years and where the nutrition interventions would be introduced after the baseline survey. Sample size was estimated based on the prevalence of maternal IFA consumption (95 tablets) and dietary diversity (51%) (national survey data [20]), the expected change after intervention (20 IFA tablets and 15 percentage points in dietary diversity), the power to detect those differences at 0.80, a level of significance at 0.05, and an intra-class correlation of 0.03, yielding a sample of 600 pregnant women (PW) and 2,000 recently delivered women (RDW) with infants under 6 months of age. Survey data from PW were used to assess factors related to dietary diversity during pregnancy, while data from RDW were used to examine the determinants of total consumption of IFA and calcium tablets throughout pregnancy. This sample was selected by a three-stage cluster sampling technique: 1) random selection of *upazilas* from matched pairs in program districts; 2) random selection of unions and villages in selected *upazilas*; and 3) selection of PW and RDW within each village using random sampling.

Data were collected via face-to-face interviews using a structured questionnaire by researchers from Data Analysis and Technical Assistance Limited (DATA), an experienced and well-qualified survey firm in Bangladesh. Survey enumerators were trained by mixed methods (lecture, role-play, mocked interview and practice) in a classroom setting and field-tested the questionnaire; revisions were made to the questionnaire based on the results of field-testing. The questionnaire was prepared initially in English and translated into Bangla, then back translated into English to double check for accuracy and consistency.

Ethical approval was obtained from the Institutional Review Boards of the BRAC University in Bangladesh and the International Food Policy Research Institute, USA. Written informed consent was obtained from all women ≥18 years. For women <18 years of age, we obtained their assent and the permission of their guardians, i.e., their parents or husbands, to participate in the study.

### Dependent variables

We constructed three primary outcomes related to maternal nutrition practices: 1) total number of IFA tablets consumed throughout the last pregnancy; 2) total number of calcium tablets consumed throughout the last pregnancy; and 3) dietary diversity during pregnancy.

RDW were asked to report how many IFA or calcium tablets they consumed during their last pregnancy. During monthly visits to PW’s homes, BRAC FHW recorded the number of IFA and calcium tablets consumed in a mother-baby book as part of the MNCH program; this book was used to assist women in their recall. Maternal dietary diversity during pregnancy was assessed among PW using an individual 24-hour diet recall, recording all foods and beverages consumed in the past 24 hours. These foods were then grouped into 10 categories [21]. A dietary diversity score was calculated as the number of food groups consumed out of 10 food groups, with a minimum of 5 food groups per day as recommended for women of reproductive age to achieve their micronutrient needs [22].

### Independent variables

**Fig 1:**
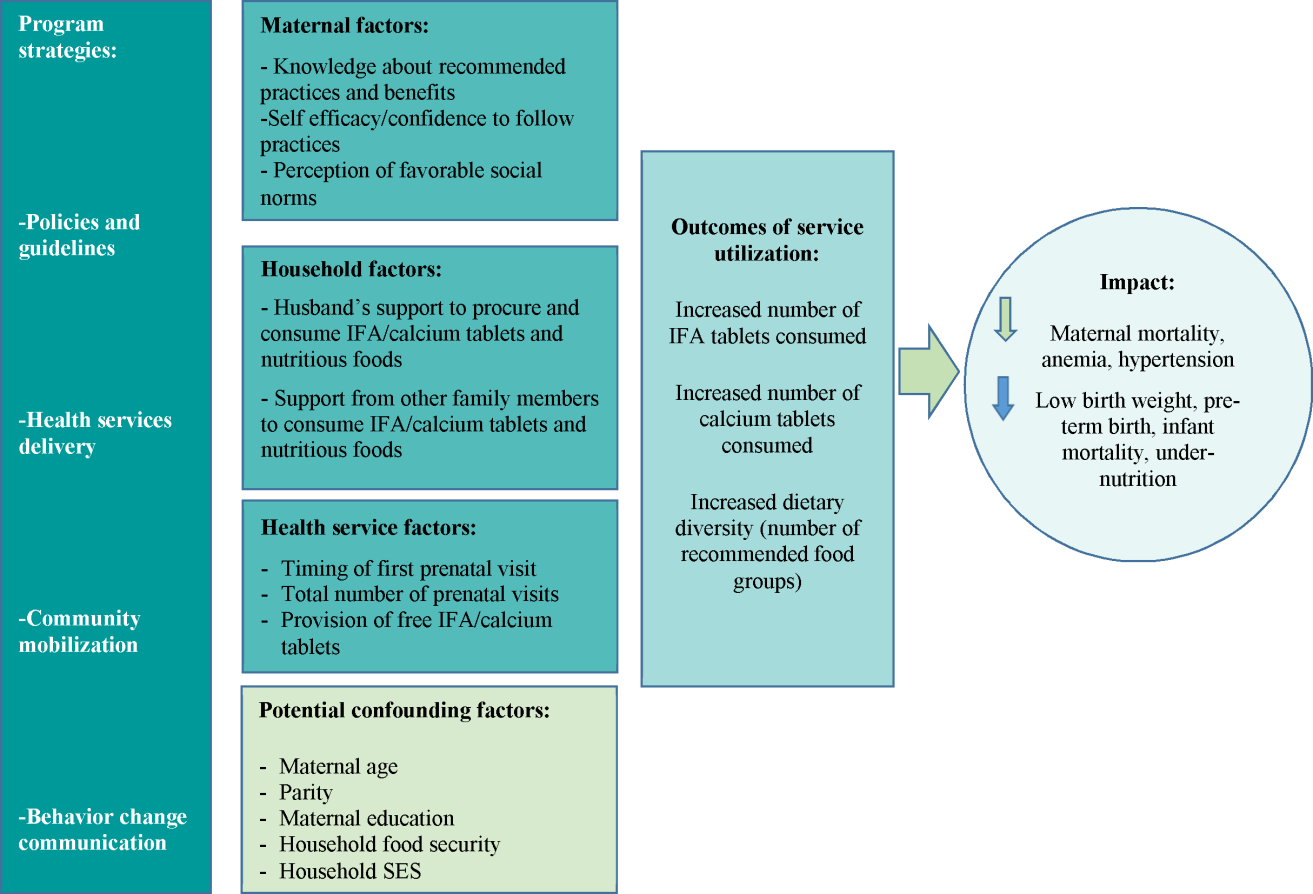
Conceptual framework of factors influencing maternal nutrition practices.

The selection of potential determinants of maternal nutrition practices was guided by a conceptual framework (Fig 1). These are the ‘exposure’ variables used to determine the predicted effects on IFA, calcium, and dietary diversity outcomes. Each of these is potentially modifiable through appropriate program design and implementation.

#### Maternal factors

*Knowledge of IFA or calcium* were assessed by asking RDW what they know about anemia, IFA and calcium supplementation, recommended numbers of IFA and calcium tablets per month and throughout pregnancy, and benefits of IFA and calcium to mothers and babies. For *knowledge of dietary diversity*, PW were asked about the benefits and importance of adequate nutrition and what foods should be eaten during pregnancy. Each knowledge item was given a score of 1 (correct) or 0 (incorrect), and the sums were used as the knowledge scores. Total scores for knowledge of IFA (range 0–10), calcium (range 0–6), and dietary diversity (range 0–15) varied, and each was categorized as low, medium or high knowledge levels based on tertiles.

*Other behavioral determinants of dietary diversity* were measured by asking women whether they agreed or disagreed with statements of belief, self-efficacy, and perceived social norms related to consuming the recommended amount and varieties of food during pregnancy (S1 Table). Each statement was given a score of 1 (agree) or 0 (disagree) with total scores ranging from 1 to 9, then the sum was divided into tertiles to obtain high, medium and low categories.

#### Household factors

*Husband’s support* was assessed by asking women whether their husbands helped to acquire or purchase diverse foods or supplements, reminded them to consume these foods or supplements, reviewed their weight gain chart and helped them find ways to gain adequate weight, and provided other support during pregnancy (S2 Table). Each statement was given a score of 1 (agree) or 0 (disagree). Total score ranged from 1 to 8, and the sum was divided into tertiles to obtain high, medium and low support categories. We also asked about support received from *other family members* during pregnancy, e.g. reminding her to take supplements.

#### Health service factors

Exposure to prenatal health visits was measured by asking about the *timing* of the first prenatal visit (early enrollment at <3 months of pregnancy, intermediate enrollment at 3–6 months, or late enrollment at >6 months) and the *total number* of visits (≤4 versus >4 visits). Because the total number of visits is influenced by when prenatal care started, we have included both variables in the models to adjust for each other. Women were also asked whether they were visited at home by FHWs and the total number of visits, and whether they received IFA/calcium supplements for free (fully throughout pregnancy) or purchased them (fully or partially during pregnancy).

### Control variables

Maternal characteristics that were examined as control variables were age, education (categorized as illiterate, elementary, middle, and high school or higher), and parity. We also controlled for household socioeconomic status (SES) and food security. An index for household SES was constructed using a principal components analysis of variables on housing conditions and asset holdings, and the first component derived from component scores was used to divide the SES score into tertiles [23,24]. Household food security was measured using the FANTA/USAID Household Food Insecurity Access Scale [25].

### Statistical analysis

Descriptive analysis was used to examine the characteristics of the study sample. Bivariate analyses were conducted to test the associations between each potential determinant with the total number of IFA or calcium tablets consumed and dietary diversity. Multivariate linear and logistic regression analyses were used to examine the association between the determinants and IFA/calcium supplement use and dietary diversity, respectively, adjusting for timing of first prenatal care and total of prenatal care visits, maternal (age, education, and parity) and household characteristics (SES and food security). Population attributable risk analysis [26] was used to estimate the additional number of IFA/calcium tablets consumed or additional proportion of PW consuming a diverse diet under different scenarios (i.e., exposure to each determinant or combination of determinants), using select modifiable factors that were identified based on the regression results. All analysis was done using Stata version 13.1 software [27]. Statistical significance was defined as p-value <0.05.

## Results

### Characteristics of the study sample

The mean age of mothers was 24 years (ranged 13–44) (Table 1). The proportion of adolescents (13–19 years) among PW was 27.0% and among RDW was 20.2%. More than 10% of the women were illiterate, and over 80% did not complete high school. Two-thirds of the women reported receiving medium or high levels of support from their husbands, but less than 10% received support from other family members for IFA or calcium consumption. During pregnancy, nearly half of the women had their first prenatal visit in the first trimester, two-thirds achieved at least four visits, and 86% of women were visited at home by FHWs with average 3.5 ± 3.1 home visits. The proportion of women who received free IFA and calcium supplements was 37.7% and 26.5%, respectively.

**Table 1:** Sample characteristics.

	Pregnant women		Recently delivered women	
	n	Percent	n	Percent
**Maternal factors:**				
Age (years)	2000	23.96 ± 5.59	2000	24.47 ± 5.51
***Education (highest grade completed)***				
Illiterate	73	12.17	232	11.60
Elementary school	189	31.50	703	35.15
Middle school	268	44.67	758	37.90
High school or higher	70	11.67	307	15.35
***Parity***				
0	220	36.67		
1	203	33.83	772	38.60
2	177	29.50	681	34.05
≥3			547	27.35
***Knowledge of IFA***				
Low	120	20.00	371	18.55
Medium	316	52.67	1115	55.75
High	164	27.33	514	25.70
***Knowledge of calcium***				
Low	138	23.00	344	17.20
Medium	280	46.67	1003	50.15
High	182	30.33	653	32.65
***Knowledge of dietary diversity***				
Low	136	22.67	—	—
Medium	286	47.67	—	—
High	178	29.67	—	—
***Other behavioral determinants of dietary diversity (enabling beliefs*, *self-efficacy*, *and social norms)***				
Low	182	30.33	—	—
Medium	203	33.83	—	—
High	215	35.83	—	—
**Household factors:**				
***Support from husband***				
Low	266	44.33	663	33.15
Medium	198	33.00	717	35.85
High	136	22.67	620	31.00
***Support from other family members for IFA***	—	—	169	8.45
***Support from other family members for calcium***	—	—	151	7.55
***Household food security***	354	59.00	1109	55.45
***Household SES (tertile)***	200	33.33	655	33.37
**Health service factors:**				
***Prenatal care timing (months of pregnancy)***				
Early (<3 months)	293	48.83	918	45.90
Intermediate (3–6 months)	307	51.17	898	44.90
Late (>6 months)	—	—	184	9.20
***Total prenatal visits (>4 visits)***	—	—	1342	67.10
***Visited home by FHWs***	440	73.30	1710	85.50
***Number of home visits by FHWs***	440	3.39 ± 2.57	1710	3.55 ± 3.15
***Received IFA tablets for free***	225	37.50	754	37.70
***Received calcium tablets for free***	130	21.67	530	26.50

### Knowledge and practices related to maternal nutrition

**Fig 2:**
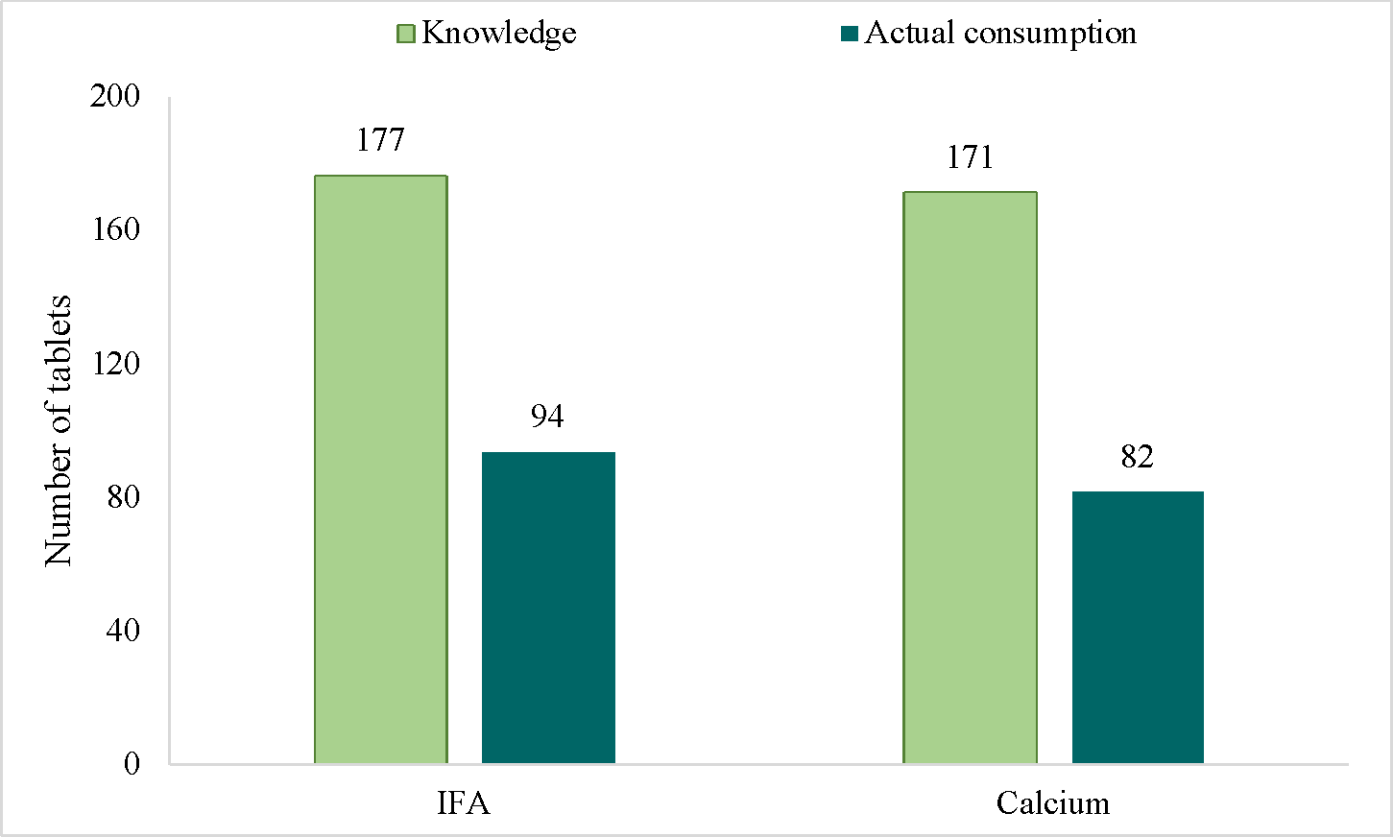
Knowledge-practice gaps for IFA/calcium supplement use among recently delivered women.

Overall, nearly 90% of RDW ever consumed IFA and calcium supplements during the last pregnancy, but the mean duration of use was only 3 months. There were large gaps between knowledge and practices related to the intake of IFA and calcium supplements. Women knew that both supplements should be consumed daily for 6 months or approximately 180 tablets in total throughout pregnancy as per the national policy guidelines. However, the reported mean numbers of tablets consumed was 94 ± 68 tablets for IFA and 82 ± 66 tablets for calcium (Fig 2).

**Fig 3:**
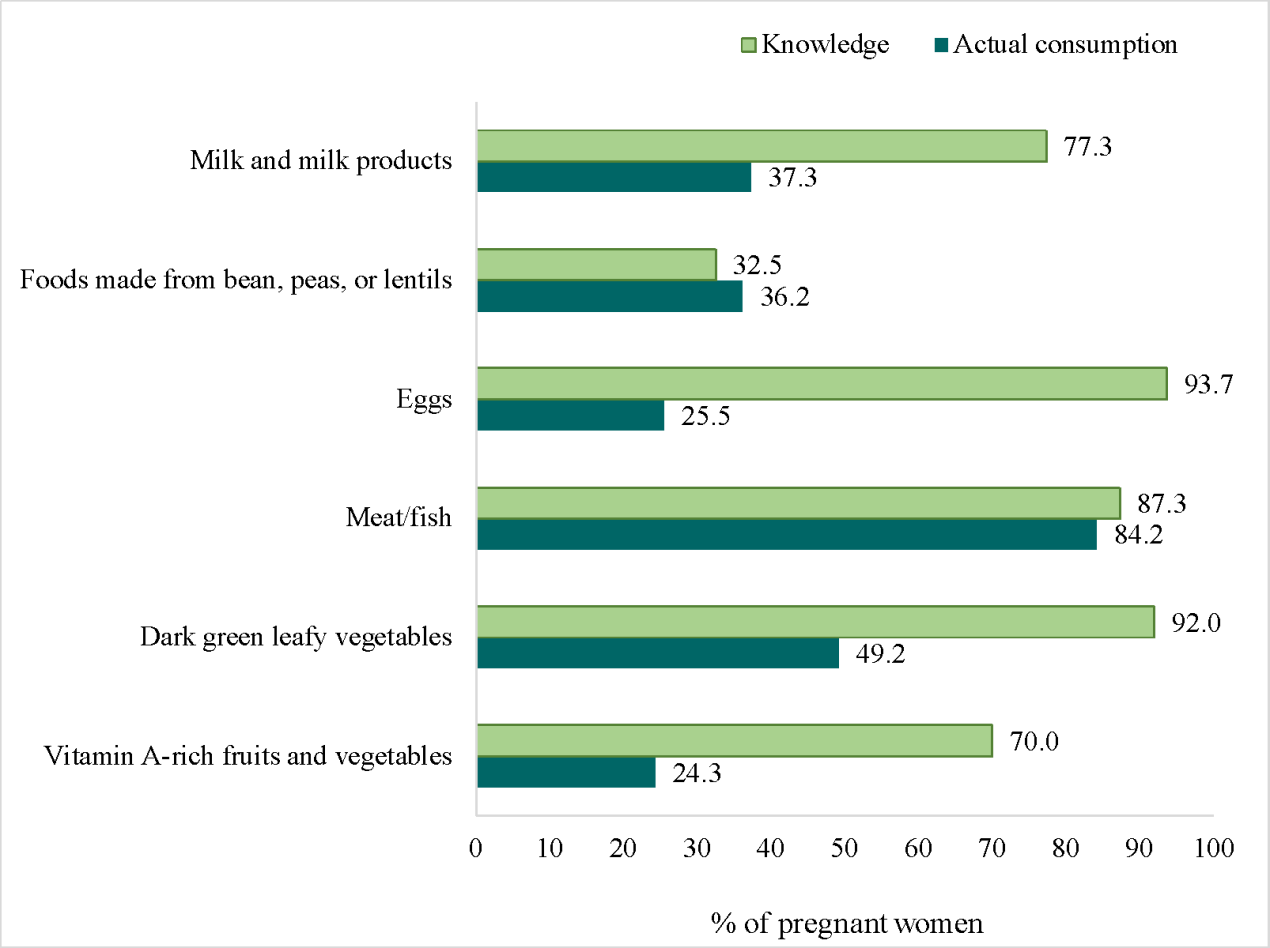
Knowledge-practice gaps for dietary diversity among pregnant women.

Similarly, large gaps were observed between knowledge and practices related to dietary diversity (Fig 3). Nearly three-quarters of PW named at least 5 food groups that should be consumed each day. However, only half of them consumed the recommended minimum of 5 food groups in the previous day. The largest knowledge-to-practice gaps were related to the consumption of eggs (94% reported eggs should be consumed daily but only 26% had consumed an egg in the previous day), milk/milk products (77% versus 37%), vitamin A-rich fruits and vegetables (70% versus 24%), and dark green leafy vegetables (92% versus 49%).

### Determinants of IFA and calcium supplement use

Maternal knowledge was strongly associated with consumption of IFA (Table 2) and calcium supplements (Table 3). Compared to women with low knowledge, those with medium knowledge consumed 19 more IFA and 23 more calcium tablets, and those with high knowledge consumed 31 more IFA and 30 more calcium tablets.

**Table 2:** Factors associated with consumption of IFA tablets.

	Bivariate		Multivariate	
	β	95% CI	β	95% CI
**Maternal factors**				
***Knowledge on IFA (low as ref)***				
Medium	30.29***	22.58, 38.01	18.58**	8.74, 28.43
High	49.23***	40.46, 58.00	30.73***	17.74, 43.72
**Household factors**				
***Support from husband (low as ref)***				
Medium	23.30***	16.36, 30.25	15.77**	7.48, 24.06
High	39.36***	32.16, 46.56	24.84***	17.96, 31.72
***Support from other family members***	9.31+	-1.34, 19.96	6.53+	-1.35, 14.41
**Health service factors**				
***Prenatal care timing (late as ref)***				
Early (<3 months)	53.46***	43.03, 63.90	27.58***	16.84, 38.33
Intermediate (3–6 months)	39.35***	28.89, 49.81	22.62***	12.30, 32.94
***Total prenatal visits (≤ 4 times as ref)***				
Adequate (> 4 times)	25.82***	19.62, 32.02	8.97*	1.12, 16.81
***Number of home visits by FHWs***	4.67***	2.77, 96.57	3.84**	1.52, 6.15
Received IFA for free	8.26**	2.16, 14.37	9.25	-2.08, 20.58
**Potential confounding factors**				
***Age (≥30 y as ref)***				
13–19.9 y	12.24*	2.85, 21.64	1.94	-10.22, 14.10
20–29.9 y	10.06*	2.35, 17.78	-0.33	-9.73, 9.08
***Education (Illiterate as ref)***				
Elementary school	13.61**	3.86, 23.36	7.99+	-1.44, 17.42
Middle school	24.54***	14.88, 34.20	10.79**	2.82, 18.76
High school or higher	56.24***	45.04, 67.43	35.13***	22.75, 47.50
***Parity (1 as ref)***				
2	-7.85*	-14.77, -0.93	5.54	-5.73, 16.81
≥ 3	-20.55***	-27.90, -13.20	4.79	-3.02, 12.59
***Household food security***	17.17***	11.25, 23.08	4.34	-1.06, 9.73
***Household SES (low as ref)***				
Medium	9.40*	2.11, 16.68	1.28	-6.60, 9.15
High	20.96***	13.68, 28.25	1.45	-7.56, 10.47

*p<0.05

**p<0.01

***p<0.001

**Table 3:** Factors associated with consumption of calcium tablets.

	Bivariate		Multivariate	
	β	95% CI	β	95% CI
**Maternal factors**				
***Knowledge on calcium (low as ref)***				
Medium	37.43***	29.66, 45.20	23.47***	13.17, 33.76
High	48.64***	40.35, 56.93	30.28***	19.32, 41.24
**Household factors**				
***Support from husband (low as ref)***				
Medium	22.86***	16.17, 29.55	13.34**	5.93, 20.75
High	42.31***	35.37, 49.25	26.32***	19.45, 33.20
***Support from other family members***	17.12**	6.27, 27.98	11.42*	1.30, 21.54
**Health service factors**				
***Prenatal care timing (late as ref)***				
Early (<3 months)	48.64***	38.48, 58.80	21.08***	11.24, 30.91
Intermediate (3–6 months)	35.85***	25.67, 46.02	17.73**	5.61, 29.86
***Total prenatal visits (≤ 4 times as ref)***				
Adequate (>4 times)	21.05***	15.00, 27.10	6.02	-1.87, 13.92
***Number of home visits by FHWs***	5.01***	3.17, 6.85	4.49***	2.44, 6.54
Received calcium for free	10.37**	3.87, 16.87	16.19**	6.78, 25.60
**Potential confounding factors**				
***Maternal age (≥30 y as ref)***				
13–19.9 y	6.36	-2.77, 15.48	-0.53	-11.92, 10.87
20–29.9 y	6.98+	-0.51, 14.48	-2.11	-9.86, 5.65
***Education (Illiterate as ref)***				
Elementary school	14.43**	5.04, 23.82	8.65*	1.54, 15.75
Middle school	26.61***	17.31, 35.92	13.89**	4.20, 23.57
High school or higher	60.38***	49.60, 71.17	43.20***	30.81, 55.59
***Parity (1 as ref)***				
2	-6.19+	-12.92, 0.54	2.01	-7.60, 11.62
≥ 3	-15.53***	-22.69, -8.38	4.11	-3.757, 11.97
***Household food security***	22.25***	16.55, 27.95	6.50*	0.44, 12.56
***Household SES (low as ref)***				
Medium	13.87***	6.84, 20.89	3.49	-3.22, 10.20
High	25.20***	18.17, 32.22	3.95	-3.97, 11.87

*p<0.05

**p<0.01

***p<0.001

Women with high support from their husbands were likely to consume more IFA and calcium tablets (25 and 26 respectively), compared to those with low support. Women who received reminders from other family members to take the supplements also consumed more IFA (6 tablets) and calcium (11 tablets).

Early initiation of prenatal visits and higher total number of visits were significantly associated with higher IFA and calcium tablet consumption. Compared to women who received their first visit later, those who enrolled early (in the first trimester) were likely to consume 26 more IFA and 21 more calcium tablets. Receiving at least 4 prenatal visits was also associated with consumption of 9 more IFA tablets. For each additional home visit by FHWs received, women were more likely to consume 4 IFA and 5 calcium tablets. Women who received IFA or calcium tablets for free appeared to have higher consumption compared to women who had to buy them, but this association remained significant in the multivariate model for calcium supplements only.

Among the control variables, higher education was significantly associated with higher consumption of both IFA and calcium tablets. In addition, women with higher numbers of previous births (3 or more) were less likely to consume IFA or calcium tablets compared to those with a first birth; however, this association was not significant in the multivariate models. Both household food security and SES were associated with consumption of IFA and calcium in the bivariate models, but only food security was associated with consumption of 6 more calcium tablets in the multivariate model.

### Determinants of dietary diversity

The three main factors significantly associated with maternal dietary diversity were maternal knowledge; other behavioral determinants (enabling beliefs, self-efficacy, and social norms); and husband’s support (Table 4). Medium and high maternal knowledge were strongly associated with higher odds of consuming ≥ 5 food groups (OR: 1.7–1.8), compared to women with low knowledge. Similar results were observed for women with high confidence, self-efficacy and perception of enabling social norms. Compared to women who received low level of support from their husbands, those with high support were nearly two times more likely to consume diverse diets. Household food security and SES were associated with consumption of diverse diets in the bivariate model, but this association did not remain significant in the fully adjusted models.

**Table 4:** Factors associated with maternal dietary diversity (≥ 5 food groups).

	Bivariate		Multivariate	
	OR	95% CI	OR	95% CI
**Maternal factors**				
***Knowledge on proper diet (low as ref)***				
Medium	2.01**	1.32, 3.05	1.72*	1.13, 2.61
High	2.21**	1.40, 3.48	1.76*	1.00, 3.12
***Other behavioral determinants (enabling beliefs*, *self- efficacy*, *and social norms—low as ref)***				
Medium	1.72**	1.15, 2.58	1.59*	1.02, 2.49
High	2.55***	1.70, 3.83	1.78*	1.09, 2.89
**Household factors**				
***Support from husband (low as ref)***				
Medium	1.46*	1.01, 2.12	1.33	0.89, 1.99
High	2.69***	1.75, 4.14	1.94*	1.12, 3.34
**Health service factors**				
***Prenatal care timing***				
Early enrollment	1.19	0.86, 1.64	1.03	0.78, 1.37
***Number of home visits by FHWs***	0.03	-0.07, 0.13	1.00	0.88, 1.12
**Potential confounding factors**				
***Age (<20 y as ref)***				
20–29 y	0.74	0.51, 1.08	0.96	0.57, 1.63
30–44 y	0.72	0.44, 1.18	1.37	0.68, 2.76
***Education (Illiterate as ref)***				
Elementary school	2.78**	1.52, 5.08	2.77**	1.38, 5.56
Middle school	3.50***	1.95, 6.27	2.68**	1.30, 5.52
High school or higher	9.53***	4.44, 20.42	6.65***	3.17, 13.92
***Parity (0 as ref)***				
1	0.78	0.53, 1.14	0.94	0.53, 1.69
≥2	0.74	0.50, 1.10	0.96	0.50, 1.82
***Household food security***	1.72**	1.24, 2.39	1.17	0.78, 1.75
***Household SES (low as ref)***				
Medium	1.84**	1.23, 2.74	1.25	0.92, 1.71
High	2.40***	1.60, 3.58	1.20	0.84, 1.73

*p<0.05

**p<0.01

***p<0.001

### Population attributable risk estimation

**Fig 4:**
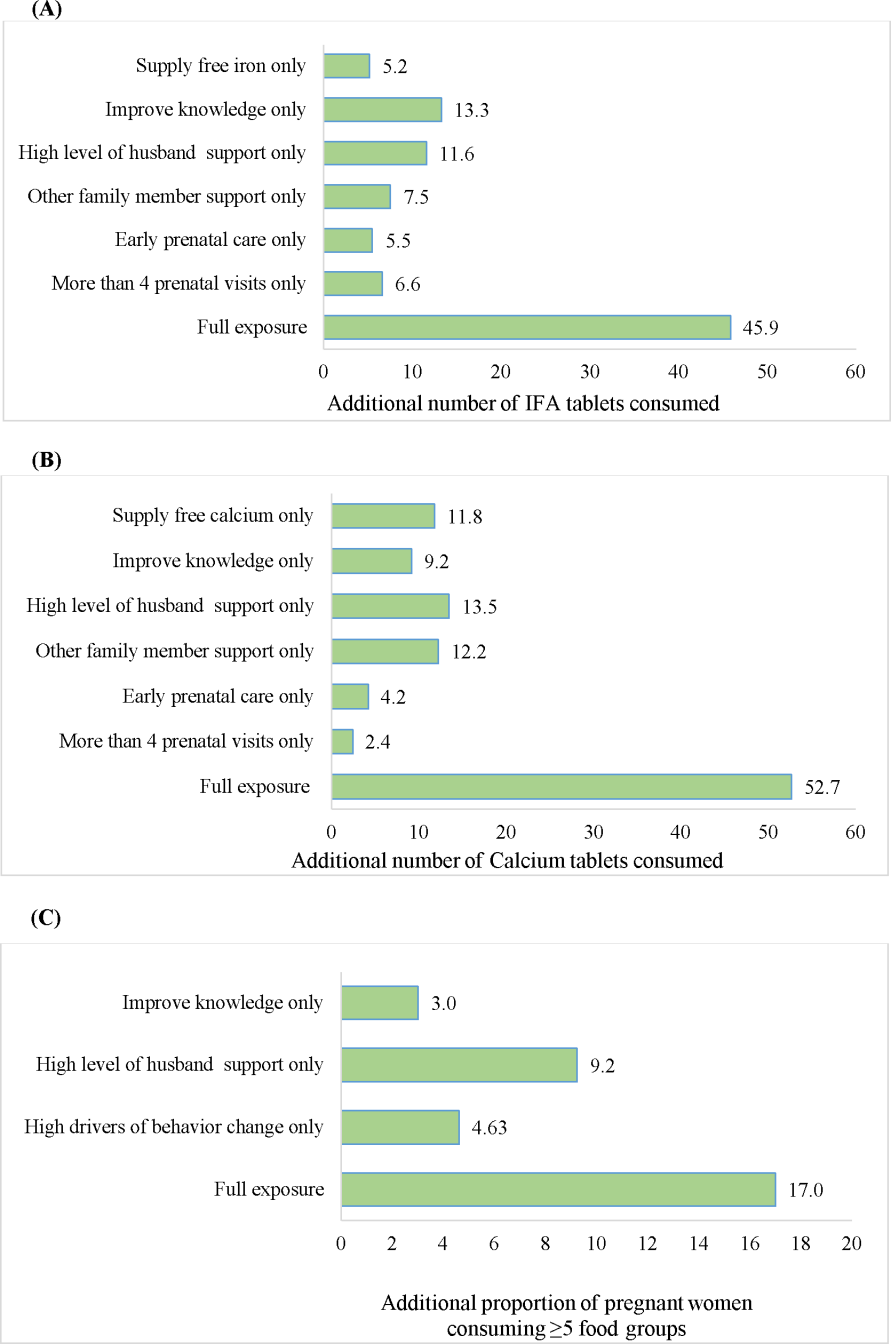
Additional consumption of IFA/calcium tablets and dietary diversity attributable to select determinant factors. (A) Additional number of IFA tablets consumed above the current average (94 tablets). (B) Additional number of calcium tablet consumed above the current average (82 tablets). (C) Additional proportion of pregnant women consuming diverse diet above the current average (50.7%).

The population attributable risk analyses indicated that under the combined conditions of good knowledge, high level of husband and family support, early initiation of prenatal visits, and at least 4 prenatal visits, women would consume an additional 46 IFA tablets and 53 calcium tablets during pregnancy (Fig 4). This would add to the current mean consumption of 94 IFA and 82 calcium tablets for a total of 140 IFA and 137 calcium tablets, respectively, thereby approaching the recommended number of tablets consumed during pregnancy. Similarly, exposure to a combination of good knowledge; high enabling beliefs, self-efficacy, and social norms; and high level of husband’s support would result in 17% more PW consuming diverse diets.

## Discussion

Our study identifies factors associated with current maternal nutrition practices and quantifies what can be achieved through a set of strategies to optimize practices. Our study contributes to a limited but growing literature, both qualitative [11,28,29] and quantitative [30–35], on the various personal, socio-cultural, or logistical factors that influence the use of IFA supplements delivered through different programs and in different contexts. We also went beyond IFA supplement to examine factors influencing the use of calcium supplements, which is a recent intervention based on the new WHO guidelines [12]. We find that several factors—maternal knowledge, family support, adequate number of prenatal visits, and free supplies play a key role in facilitating the consumption of both IFA and calcium supplements.

In addition to micronutrient supplement use, we include dietary diversity as an essential maternal nutrition practice. We observed that health service factors such as prenatal visits are not associated with dietary diversity, but maternal factors (knowledge, beliefs, self-efficacy, and social norms) are prominent factors together with husband’s support. Thus, strengthening the performance of FHWs in improving both knowledge and other drivers of behavior change (e.g. PW’s self-efficacy and perception of social norms) to close the knowledge-practice gaps, and engaging husbands and other family members to provide support to women can lead to large improvements in maternal nutrition practices.

Through attribution analyses, we observed that exposure to all these factors together could have an additive effect on maternal nutrition practices, with potentially 68% of PW achieving the minimum dietary diversity and total consumption of 140 IFA and 135 calcium tablets throughout pregnancy, bringing women closer to the recommendations. This suggests that a combination of selected strategies could be used to increase maternal practices in the context of a well-functioning and high-coverage MNCH program.

In our study context, we observed high exposure to prenatal visits, intake of any IFA and calcium tablets, and awareness about dietary diversity. However, women only partly achieved the recommended behaviors (e.g. consumed 90 tablets compared to the recommended 180 tablets. Good knowledge alone did not naturally translate into behavior change, especially without the support from husbands and family members. Previous qualitative work in Bangladesh reported that husbands had little influence on decisions regarding pregnancy care while mothers-in law or older people played a greater role [28], but we observed important supportive roles of both husbands and other family members. Women who reported a high level of husband’s support were more likely to consume IFA and calcium tablets and diverse diet. In Bangladesh, men traditionally grow and/or purchase the food and are considered the most important decision-makers involving money spending [28], so they often determine what the family eats. Our findings corroborate other study results that documented the influence of husband and family support on micronutrient supplement adherence in Peru [36], Zimbabwe [37] and Kenya [38]. Thus, increasing husband’s and family support to procure and ensure adequate supplements and foods is critical. Furthermore, maternal nutrition practice is not a one-off behavior and requires sustained behavior change throughout the pregnancy period, so addressing simultaneously the relevant factors that influence the different facets of behavior change is likely needed to achieve desired health outcomes.

Both IFA and calcium supplementation is recommended during pregnancy, particularly in the settings with low intake of iron and calcium and high prevalence of anemia and/or pre-eclampsia. As in many other countries, IFA supplementation in Bangladesh is designed to be delivered through ANC that functions within the context of the government health-care system [39]. However, there have been obstacles related to the procurement and distribution of IFA supplements, and many PW do not receive or purchase IFA during ANC [39]. Our findings show that providing free IFA and calcium was associated with higher IFA and calcium consumption. Given that FHWs as part of the MNCH program visit PW’s homes on a regular basis, distributing micronutrient supplements at the doorsteps of women’s homes could be feasible and would ensure that women receive adequate supplements [8,34]. Recent evidence also suggests the advantages in replacing IFA supplements with multiple micronutrient supplements in populations experiencing multiple co-existing micronutrient deficiencies [40], there is the potential of reducing the regimen in the future, from separate IFA and calcium tablets to a single table [40]. However, given the potential of calcium to inhibit iron absorption in the gastrointestinal tract [41,42], special considerations may be needed such as lowering the dosage of iron [43] and calcium [19], and increasing vitamin C to enhance iron absorption.

Improving the coverage of proven maternal nutrition interventions on a large scale in high-need settings can substantially reduce maternal and neonatal mortality and morbidity [2]. MNCH programs provide a delivery platform that can potentially reach large proportions of PW with essential nutrition interventions. However, effective integration of a package of nutrition interventions may require priority strategies that focus on how best to ultimately improve both delivery and utilization. Our study illustrates that improving both demand side (such as maternal knowledge, self-efficacy and perceptions of social norms) and supply side (such as early registration in prenatal care and provision of free supplements), together with family support, have the potential to achieve high maternal nutrition practices.

This study had several strengths. We utilized representative datasets of both PW and RDW from areas where the MNCH program has been active for over five years, thus providing information under conditions of routine MNCH services. We explored multiple factors related to the women, their households, and health services, to identify and highlight factors that may be modified by the MNCH program to increase maternal nutrition practices. Modeling the effects of individual and combination of factors that are positively associated with improved practices provides further evidence to advocate for investments in key strategies to increase the uptake of evidence-based maternal nutrition interventions through MNCH services.

Our study also had a few limitations and caveats to interpretation. IFA and calcium intake were self-reported by RDW up to six months after delivery and thereby may suffer from recall bias by pregnancy outcomes as well as social desirability bias since these behaviors are recommended as part of MNCH program. However, we tried to aid recall by referring to the mother-baby books, where available. As our analysis is based on a cross-sectional survey, we cannot be certain of causality and, therefore, do not claim causal attributions. Also, we emphasize that this study was carried out in the context of a well-functioning and robust MNCH program platform that delivered various services related to reproductive health and pregnancy care targeted to mothers as well as essential services related to neonatal and child health; analyses of drivers of utilization in other less well-established program platforms could likely reveal a different set of factors.

## Conclusions

Our study provides evidence of potential factors to strengthen maternal nutrition practices within MNCH programs, particularly improving knowledge, self-efficacy and perceptions of social norms among PW, and increasing husbands’ support, ensuring early registration in prenatal care, and provision of free supplements. In the context of BRAC’s MNCH program, the insights from this analysis will be applied to strengthen the delivery and uptake of IFA and calcium supplementation and to provide counseling to all family members about dietary diversity in pregnancy. Improving the delivery and uptake of these interventions has significant potential to improve maternal micronutrient deficiencies, thereby reducing the high burden of malnutrition and saving the lives of Bangladeshi mothers and children.

## Supporting information

S1 Table Questions used to assess behavioral determinants (belief, self-efficacy and social norms). (DOCX)

S2 Table Questions used to assess husbands’ support. (DOCX)

S1 Data Dataset. (XLSX)
