# Supplementary material for: Factors influencing maternal nutrition practices in a large scale maternal, newborn and child health program in Bangladesh
Source: PLoS One. 2017 Jul 10;12(7):e0179873. doi: 10.1371/journal.pone.0179873 (PMC5503174; doi:10.1371/journal.pone.0179873)
Supplement: S1 Table — (DOCX) [file pone.0179873.s001.docx]

**S1 Table. Questions used to assess behavioral determinants (belief, self-efficacy and social norms)^1^**

Please tell us if you agree with the following statements (1=agree, 0=disagree)

| My consuming right types and amount of food during pregnancy is extremely important for my health and my unborn child |
| --- |
| My consuming right types and amount of food during pregnancy is extremely important for my unborn child’s brain/education and ability to earn |
| I can manage to follow the recommendations of 5 varieties of food to be consumed during pregnancy |
| I can manage to follow the recommendations of adequate amounts of food to be consumed during pregnancy |
| My family members and community people will be angry if I consume the right types and amounts of food during pregnancy^2^ |
| I cannot consume the recommended types and amounts of food as we are poor people^2^ |
| It is too costly to obtain the recommended types and amounts of foods for my consumption during pregnancy^2^ |
| It is a good use of our family’s money to ensure the right types and amounts of foods during pregnancy and it contributes to the future welfare of the child and family |
| In my family and community, I am expected to consume so many varieties and such large amount during pregnancy |

^1^Behavioral determinants were assessed based on mothers agree or disagree to the questions. Each item was given a score of 1 (agree) or 0 (disagree). Range score: 0-9

^2^Questions with reverse coded.
