# Supplementary material for: Factors influencing maternal nutrition practices in a large scale maternal, newborn and child health program in Bangladesh
Source: PLoS One. 2017 Jul 10;12(7):e0179873. doi: 10.1371/journal.pone.0179873 (PMC5503174; doi:10.1371/journal.pone.0179873)
Supplement: S2 Table — (DOCX) [file pone.0179873.s002.docx]

**S2 Table. Questions used to assess husbands’ support^1^**

Please tell us if you agree with the following statements (1=agree, 0=disagree)

| My husband does not purchase diversified nutritious foods and does not ensure that I have these foods available^2^ |
| --- |
| My husband reminds and encourages me to consume the recommended quantity of diversified foods daily |
| My husband helps me to ensure that there are enough tablets of IFA and Calcium at home |
| My husband reminds me to take one tablet of IFA and on tablet of Calcium daily |
| My husband does not remind /helps me to take rest for 2 hours during the day in addition to sleeping at night^2^ |
| My husband and family members make me lifting heavy work load during pregnancy |
| My husband reviews my weight gain chart and helps me find ways to gain enough weight during pregnancy |
| My husband calls the health worker on mobile if I have any difficulties to do any of the above |

^1^Support from husband were assessed based on mothers agree or disagree to the questions. Each item was given a score of 1 (agree) or 0 (disagree). Range score: 0-8

^2^Questions with reverse coded.
